# Clinical characteristics, management, and prognosis of pembrolizumab-induced hypophysitis: retrospective analysis based on case reports

**DOI:** 10.3389/fimmu.2026.1823373

**Published:** 2026-06-10

**Authors:** Jingjing Huang, Junlong Cai, Wei Cheng, Yan Wang

**Affiliations:** 1 Department of Endocrinology and Metabolism, The Central Hospital of Xiangtan (The Affiliated Hospital of Hunan University), Xiangtan, China; 2 Department of Student Affairs, Hunan University of Medicine General Hospital, Huaihua, China

**Keywords:** hormone replacement therapy, hypophysitis, immune-related adverse event, management, pembrolizumab, pituitary dysfunction

## Abstract

**Background:**

Pembrolizumab-induced hypophysitis is a rare but clinically important immune-related adverse event. Its clinical features and long-term outcomes are not fully defined. This study aimed to summarize the clinical characteristics, management, and prognosis of pembrolizumab-associated hypophysitis to support early recognition and appropriate treatment.

**Methods:**

PubMed, EMBASE, Web of Science, WanFang Data, and CNKI were searched for reports of pembrolizumab-induced hypophysitis published up to Jan 31, 2026, using relevant keywords and Boolean operators. Eligible case reports and case series were screened, and patient-level data were extracted using a standardized form. Study quality was assessed with the Joanna Briggs Institute (JBI) Critical Appraisal Checklist for Case Reports.

**Results:**

A total of 22 patients were included. The median age was 66 years (range 39–85), with equal distribution between males and females. The median time to onset was 15 weeks (range 2–36), most frequently occurring within 11–20 weeks. Fatigue was the predominant symptom (86.4%), followed by nausea/vomiting (54.5%), adrenal insufficiency manifestations (40.9%), hyponatremia (31.8%), and headache (27.3%). Median cortisol and ACTH levels were markedly reduced at 0.9 μg/dL and 5.0 pg/mL, respectively. Among patients with available pituitary MRI data, normal findings were observed in 50.0% of cases, whereas pituitary enlargement was present in 27.8%. Hormone replacement therapy, primarily hydrocortisone, was administered in 81.8% of cases. Clinical improvement occurred in 90.9% of patients; however, persistent endocrine dysfunction remained in 95.5%, with recovery of the hypothalamic–pituitary axis documented in only one case. Rechallenge was performed in seven patients without recurrence in most cases. According to the WHO-UMC system, 86.4% of cases were classified as probable.

**Conclusion:**

Pembrolizumab-induced hypophysitis typically presents subacutely with nonspecific symptoms and frequently exhibits normal imaging findings. Although clinical symptoms improve with prompt hormone replacement, permanent endocrine insufficiency is common. Early recognition and long-term endocrine follow-up are essential in affected patients.

## Introduction

Immune checkpoint inhibitors (ICIs) have reshaped the therapeutic landscape of multiple advanced malignancies by restoring antitumor immune surveillance through blockade of inhibitory signaling pathways (1). Pembrolizumab, a monoclonal antibody targeting programmed cell death protein 1 (PD-1), enhances T-cell activation by preventing interaction with its ligands PD-L1 and PD-L2, thereby reversing tumor-induced immune suppression (2). Since its approval, pembrolizumab has demonstrated substantial survival benefits across a broad spectrum of cancers, including non-small cell lung cancer, melanoma, urothelial carcinoma, and gynecologic malignancies (3).

However, disruption of immune tolerance inevitably predisposes patients to immune-related adverse events (irAEs), which can involve nearly every organ system (4–7). Endocrine irAEs are among the most frequently encountered toxicities and include thyroiditis, adrenal insufficiency, hypophysitis, and immune-mediated diabetes (8). While thyroid dysfunction is relatively common, hypophysitis remains an uncommon but clinically significant complication. In contrast to cytotoxic T-lymphocyte–associated antigen 4 (CTLA-4) inhibitor–induced hypophysitis, which is often characterized by pituitary enlargement and multiple hormonal deficiencies, PD-1 inhibitor–associated hypophysitis appears less frequent, more insidious in onset, and frequently presents with isolated adrenocorticotropic hormone (ACTH) deficiency and normal pituitary imaging (8). The pathophysiology of PD-1–related hypophysitis remains incompletely understood. Unlike CTLA-4, PD-1 and PD-L1 are not strongly expressed on normal pituitary tissue, suggesting a distinct immunologic mechanism. Proposed pathways include indirect autoimmune activation, cross-reactive T-cell responses, and the generation of antipituitary antibodies, although serologic evidence remains inconsistent (9). Moreover, aging-associated immune dysregulation and enhanced systemic inflammatory responses may further increase susceptibility to endocrine irAEs (10). These differences underscore that PD-1–induced hypophysitis represents a clinically and immunologically distinct entity from CTLA-4–associated disease. Clinically, pembrolizumab-induced hypophysitis often presents with nonspecific symptoms such as fatigue, anorexia, nausea, headache, hypotension, or hyponatremia (11). These manifestations frequently overlap with cancer-related symptoms, infection, or treatment-related toxicity, which can delay diagnosis (12). Notably, delayed diagnosis can result in adrenal crisis, prolonged hospitalization, or life-threatening complications. Although hormone replacement therapy typically leads to rapid symptomatic improvement, recovery of pituitary function appears uncommon, and many patients require lifelong glucocorticoid replacement (13).

Despite increasing clinical awareness, current knowledge of pembrolizumab-induced hypophysitis is largely derived from individual case reports and small case series. The true clinical spectrum, imaging characteristics, treatment strategies, endocrine recovery patterns, and long-term outcomes remain incompletely defined. In addition, questions remain regarding immune checkpoint inhibitor continuation, rechallenge safety, and oncologic outcomes after endocrine irAEs (8). Therefore, we conducted a retrospective, case-based analysis of published reports to systematically characterize the clinical features, laboratory and radiologic findings, management approaches, and prognosis of pembrolizumab-induced hypophysitis. By synthesizing available evidence, this study aims to enhance recognition of this underdiagnosed endocrine complication and to provide practical insights for optimized long-term management in patients receiving PD-1 blockade.

## Methods

### Study design and search strategy

A comprehensive literature search was conducted in PubMed, Embase, Web of Science, Wanfang Data, and China National Knowledge Infrastructure (CNKI) to identify reports of pembrolizumab-induced hypophysitis published up to Jan 31, 2026. The search strategy incorporated both controlled vocabulary (MeSH terms) and free-text terms. Specifically, we used MeSH terms included “pembrolizumab,” OR “anti–PD-1,” OR “PD-1 inhibitor,” OR “immune checkpoint inhibitor,” combined with “hypophysitis,” OR “pituitary dysfunction,” OR “pituitary inflammation,” OR “hypopituitarism,” OR “immune-related adverse event.” Reference lists of eligible articles and relevant reviews were also manually screened to identify additional reports.

### Inclusion and exclusion criteria

We included case reports and case series describing hypophysitis attributed to pembrolizumab and providing sufficient patient-level clinical information for data extraction. Reports were excluded if they were review articles, mechanistic studies, animal experiments, conference abstracts without extractable data, or lacked adequate clinical details to support analysis. Duplicate records were removed electronically and manually verified. Potentially overlapping reports were assessed using key publication and patient-level details, and only the most complete report was retained to avoid duplicate counting. Pembrolizumab-induced hypophysitis was defined as new-onset pituitary dysfunction temporally associated with pembrolizumab exposure, supported by compatible clinical manifestations and biochemical evidence of central adrenal insufficiency and/or other pituitary hormone deficiencies, with or without pituitary MRI abnormalities. For patients with normal MRI findings, the diagnosis was considered acceptable when low morning cortisol was accompanied by inappropriately low or normal ACTH levels, clinical features consistent with adrenal insufficiency, and no more plausible alternative explanation, such as primary adrenal disease, pituitary metastasis, prior pituitary surgery, cranial radiotherapy, or exogenous glucocorticoid exposure.

### Study selection and data extraction

Two investigators independently screened titles and abstracts, followed by full-text review of potentially eligible studies. Disagreements were resolved through discussion until consensus was reached. A standardized data collection form was used to extract patient-level information, including demographic characteristics (age, sex, country), primary malignancy, pembrolizumab regimen, time to onset, clinical manifestations, laboratory findings (cortisol, ACTH, thyroid function, serum sodium), pituitary imaging results, management strategies, ICI continuation status, clinical outcomes, hormonal recovery, tumor response, and rechallenge data.

### Quality assessment of case reports

The methodological rigor of the included case reports and case series was evaluated using the Joanna Briggs Institute (JBI) Critical Appraisal Checklist for Case Reports, which comprises eight assessment domains. Two reviewers independently assessed each item as “Yes,” “No,” “Unclear,” or “Not applicable,” and any discrepancies were resolved through discussion with a third reviewer to achieve consensus.

### Causality assessment

The association between pembrolizumab and hypophysitis was evaluated using the World Health Organization–Uppsala Monitoring Centre (WHO-UMC) causality assessment system, categorizing cases as “certain,” “probable,” or “possible” based on temporal relationship, response to drug withdrawal, and exclusion of other causes.

### Statistical analysis

Descriptive statistics were performed using SPSS version 24.0. Continuous variables were summarized as medians with ranges (minimum-maximum), and categorical variables were expressed as frequencies and percentages.

## Results

### Study selection

**Figure 1:**
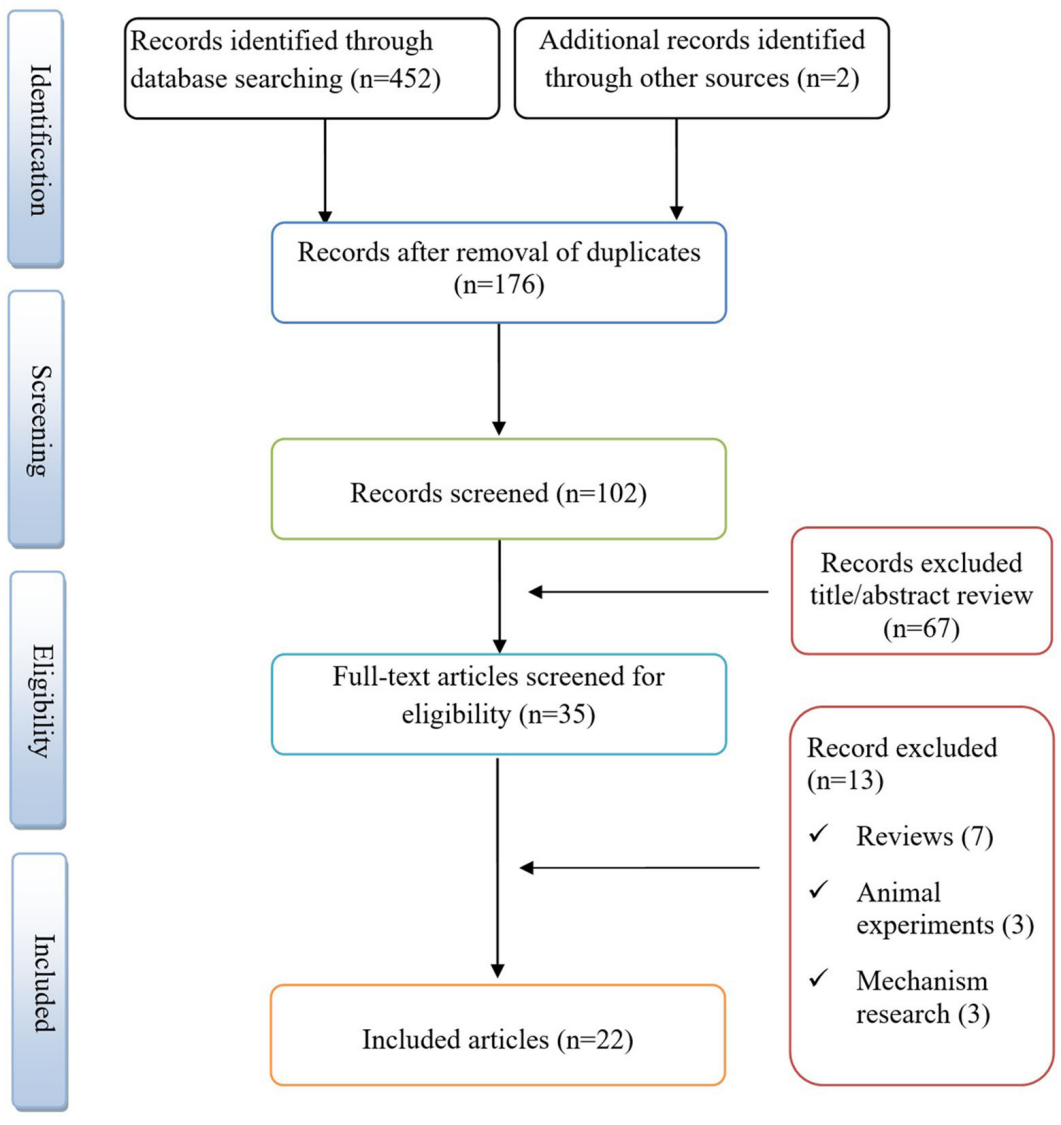
Flowchart illustrating the study selection process for inclusion.

A total of 452 records were identified through database searches, with an additional two records obtained from other sources (shown in **Figure 1**). After removal of duplicates, 176 records remained for screening. Following title and abstract review, 67 records were excluded, and 35 full-text articles were assessed for eligibility. 13 articles were excluded (7 reviews, 3 animal studies, and 3 mechanistic studies), resulting in 22 eligible articles for final inclusion (12–33). These reports described 22 patients with pembrolizumab-induced hypophysitis, whose baseline characteristics are summarized in **Supplementary Table 1**. Overall, the methodological quality of the included reports was acceptable across the eight domains of the JBI Critical Appraisal Checklist. Most cases clearly reported patient demographics, clinical manifestations, diagnostic findings, management strategies, and outcomes. Detailed item-level quality assessments are presented in **Supplementary Table 2**.

### Basic characteristics

As shown in **Table 1**, the overall cohort included 22 patients, the median age of the included patients was 66 years (range 39–85), with an equal distribution between males and females (50.0% each). Cases were reported from multiple countries, most frequently the United States (45.5%), followed by Japan (22.7%), while China, the United Kingdom, Turkey, Belgium, Spain, Australia, and Italy each contributed individual cases. The most common underlying malignancy was lung cancer (40.9%), followed by melanoma (18.2%) and urothelial/ureteral carcinoma (18.2%). Endometrial or uterine cancer accounted for 9.1% of cases, while renal cell carcinoma, breast cancer, and other malignancies were each reported in single patients. The median time from initiation of pembrolizumab to onset of hypophysitis was 15 weeks (range 2–36). Most cases occurred within 11–20 weeks (47.4%), followed by 1–10 weeks (36.8%), indicating a predominantly early-to-intermediate onset pattern. The most frequently reported dosing regimen was 200 mg every 3 weeks. Concomitant therapies were documented in 31.8% of patients and included carboplatin, paclitaxel or nab-paclitaxel, cisplatin, pemetrexed, ipilimumab, gemcitabine, trastuzumab, bevacizumab, and tocilizumab.

**Table 1:** Basic information of 22 patients with pembrolizumab-induced hypophysitis.

Parameter	Classification	Value
Gender (22)^a^	Male	11 (50.0%)
	Female	11 (50.0%)
Age (22)^a^	Years	66 (39, 85)^b^
Country (22)^a^	USA	10 (45.5%)
	Japan	5 (22.7%)
	China	1 (4.5%)
	UK	1 (4.5%)
	Turkey	1 (4.5%)
	Belgium	1 (4.5%)
	Spain	1 (4.5%)
	Australia	1 (4.5%)
	Italy	1 (4.5%)
Symptom onset time (19)^a^	Weeks	15 (2, 36)^b^
	1–10	7 (36.8%)
	11–20	9 (47.4%)
	21–30	1 (5.3%)
	31–36	2 (10.5%)
Indication (22)^a^	Lung cancer	9 (40.9%)
	Melanoma	4 (18.2%)
	Urothelial/Ureteral cancer	4 (18.2%)
	Endometrial/Uterine cancer	2 (9.1%)
	Renal cell carcinoma	1 (4.5%)
	Breast cancer	1 (4.5%)
	Other cancers	1 (4.5%)
Disease history (16)^a^	Hypertension, Type 2 diabetes mellitus, chronic kidney disease, coronary artery disease, hyperlipidemia	16 (72.7%)
Pembrolizumab dosage (21)^a^	200 mg every 3 weeks	6 (28.6%)
	200 mg (unspecified frequency)	5 (23.8%)
	400 mg every 6 weeks	1 (4.8%)
	400 mg (unspecified frequency)	1 (4.8%)
	Weight-based dosing (2 mg/kg)	2 (9.5%)
	Other dosage	6 (28.6%)
Concomitant medications (7)^a^	Carboplatin, paclitaxel/nab-paclitaxel, cisplatin, pemetrexed, ipilimumab, gemcitabine, trastuzumab, bevacizumab, tocilizumab	7 (31.8%)

^a^ Represents the number of patients with this parameter out of 22 patients; ^b^Median (minimum, maximum).

### Clinical manifestations

The clinical features of the 22 patients are summarized in **Table 2**. The most common symptom was fatigue or malaise, reported in 19 patients (86.4%). Gastrointestinal manifestations, including nausea, vomiting, and anorexia, were observed in 12 patients (54.5%). Signs suggestive of adrenal insufficiency were documented in 9 cases (40.9%), and hyponatremia occurred in 7 patients (31.8%). Headache was reported in 6 patients (27.3%). Other manifestations included weight loss (18.2%), gastrointestinal symptoms such as abdominal discomfort or diarrhea (18.2%), and dizziness or syncope (13.6%). Additional nonspecific symptoms, including myalgia, fever, hot flashes, itching, low libido, and atrial fibrillation, were described in 6 patients (27.3%).

**Table 2:** Clinical manifestations and laboratory findings of 22 patients with pembrolizumab-induced hypophysitis.

Parameter	Classification	Value
Clinical symptoms (22)^a^	Fatigue/malaise	19 (86.4%)
	Nausea/vomiting/anorexia	12 (54.5%)
	Hyponatremia	7 (31.8%)
	Headache	6 (27.3%)
	Adrenal insufficiency signs	9 (40.9%)
	Weight loss	4 (18.2%)
	Gastrointestinal symptoms	4 (18.2%)
	Dizziness/syncope	3 (13.6%)
	Other symptoms: Myalgia, fever, Hot flashes, itching, low libido, atrial fibrillation	6 (27.3%)
Pituitary MRI (18)^a^	Pituitary enlargement/swelling	5 (27.8%)
	Stalk thickening/Infundibulum enhancement	3 (16.7%)
	Inhomogeneous/Contrast enhancement	4 (22.2%)
	Other findings: Empty sella, pituitary microadenoma	3 (16.7%)
	Normal MRI findings	9 (50.0%)
Laboratory tests		
Cortisol (19)^a^	*Ref*: morning, 5–25 μg/dL	0.9 (0.4, 7.6)^b^
ACTH (19)^a^	*Ref*: morning, 7.2–63.3 pg/mL	5.0 (1.0, 16.6)^b^
TSH (10)^a^	*Ref*: 0.5–5.0 mIU/L	0.26 (0.01, 4.46)^b^
FT4 (6)^a^	*Ref*: 0.7–1.9 ng/dL	0.91 (0.60, 5.10)^b^
PRL (2)^a^	*Ref*: sex- and laboratory-dependent	17.55 (12.4, 22.7)^b^
Sodium (15)^a^	*Ref*: 135–145 mmol/L	130.0 (119.0, 143.0)^b^

^a^ Represents the number of patients with this parameter out of 22 patients; ^b^Median (minimum, maximum).

ACTH, Adrenocorticotropic Hormone; TSH, Thyroid-Stimulating Hormone; FT4, Free Thyroxine; PRL, Prolactin.

### Laboratory findings

Pituitary magnetic resonance imaging (MRI) findings were available in 18 patients. Pituitary enlargement or swelling was detected in 5 cases (27.8%), stalk thickening or infundibular enhancement in 3 (16.7%), and inhomogeneous or contrast enhancement in 4 (22.2%). Other abnormalities, such as empty sella or pituitary microadenoma, were observed in 3 patients (16.7%). Notably, normal pituitary MRI findings were observed in a substantial proportion of patients despite biochemical evidence of central adrenal insufficiency. This finding suggests that pembrolizumab-induced hypophysitis may be radiologically occult and that the absence of pituitary enlargement or stalk thickening does not exclude the diagnosis. Laboratory values were extracted from individual case reports and summarized among patients with available data. Serum cortisol and ACTH were reported in 19 patients and were markedly reduced, with median values of 0.9 μg/dL (range, 0.4–7.6) and 5.0 pg/mL (range, 1.0–16.6), respectively. Thyroid-axis parameters were less consistently reported, with median TSH and FT4 values of 0.26 mIU/L (range, 0.01–4.46) and 0.91 ng/dL (range, 0.60–5.10), respectively. Serum sodium values were available in 15 patients, with an overall median of 130.0 mmol/L (range, 119.0–143.0). Among these patients, 7 had hyponatremia, defined as serum sodium <135 mmol/L.

### Treatment and outcome

Treatment strategies and clinical outcomes are summarized in **Table 3**. Glucocorticoid replacement was the cornerstone of management. Hydrocortisone or cortisone acetate was administered in 18 patients (81.8%), while prednisone was used in 5 cases (22.7%). Thyroid hormone replacement with levothyroxine was initiated in 10 patients (45.5%), and fludrocortisone was prescribed in 2 cases (9.1%). To improve clinical interpretability, treatments were further classified in **Supplementary Table 1** as physiologic hormone replacement or high-dose corticosteroid therapy. Physiologic hormone replacement was the predominant approach, whereas explicitly reported high-dose corticosteroid therapy was documented in only 3 patients. Supportive measures, including intravenous hydration, were reported in 2 patients (9.1%). Information regarding ICI management was available in six cases. Pembrolizumab was permanently discontinued in 3 patients (50.0%), temporarily interrupted in 2 (33.3%), and continued in 1 patient (16.7%). Overall, 20 patients (90.9%) experienced clinical improvement following hormone replacement therapy, whereas 2 patients (9.1%) died due to cancer progression. Tumor response data were available in 13 patients, among whom 11 (84.6%) achieved stable disease or objective response, and 2 (15.4%) experienced progression or relapse. Notably, hormonal recovery was uncommon. Only 1 patient (4.5%) demonstrated recovery of pituitary axis function, while persistent endocrine deficiency was observed in 21 patients (95.5%). Rechallenge with pembrolizumab was reported in 7 cases; 5 patients (71.4%) tolerated re-exposure without recurrence of hypophysitis, whereas outcomes were unspecified in 2 cases (28.6%). According to the WHO–UMC causality assessment, 19 cases (86.4%) were categorized as “probable” and 3 (13.6%) as “possible,” supporting a strong association between pembrolizumab exposure and the development of hypophysitis.

**Table 3:** Treatment and prognosis of 22 patients with pembrolizumab-induced hypophysitis.

Parameter	Classification	Value
Treatment (22)^a^	Hydrocortisone/Cortisone acetate	18 (81.8%)
	Prednisone	5 (22.7%)
	Levothyroxine	10 (45.5%)
	Fludrocortisone	2 (9.1%)
	Hydration	2 (9.1%)
ICI management (6)^a^	Discontinued/stopped	3 (50.0%)
	interrupted/hold	2 (33.3%)
	continued	1 (16.7%)
Outcome (22)^a^	Clinical improvement	20 (90.9%)
	Death (cancer progression related)	2 (9.1%)
Tumor response (13)^a^	Stable disease/Response	11 (84.6%)
	Progression / Relapse	2 (15.4%)
Hormonal recovery (22)^a^	Axis recovered	1 (4.5%)
	Persistent endocrine deficiency	21 (95.5%)
Rechallenge (7)^a^	No recurrence	5 (71.4%)
	Unspecified	2 (28.6%)
WHO-UMC causality category (22)^c^	Probable	19 (86.4%)
	Possible	3 (13.6%)

^a^ Represents the number of cases describing this parameter out of 22 patients; ^b^Median (minimum, maximum)

^c^ Under the WHO-UMC system, a “certain” case shows a clear temporal relationship with drug use, improvement upon withdrawal, and recurrence upon rechallenge. A “probable” case has a reasonable time relationship, is unlikely explained by other causes, and improves after withdrawal without requiring rechallenge. A “possible” case has a reasonable time relationship but may also be due to other conditions, and the effect of withdrawal is unclear.

## Discussion

Pembrolizumab-induced hypophysitis is rare but clinically significant, representing an underrecognized endocrine immune-related adverse event in the era of expanding PD-1 blockade (28). In this retrospective analysis, we systematically characterized the clinical features, endocrine phenotype, management strategies, and outcomes of pembrolizumab-induced hypophysitis. Several key observations emerged. ACTH deficiency was the most consistently documented hormonal abnormality, pituitary imaging was frequently normal, hormone replacement led to rapid symptomatic improvement, and recovery of corticotropic function was uncommon. However, endocrine evaluation was not uniform across the included reports, and thyroid, prolactin, gonadal, and growth hormone axes were variably or incompletely assessed. Therefore, the predominance of isolated ACTH deficiency should be interpreted cautiously in the context of incomplete evaluation of other pituitary axes. In addition, pembrolizumab rechallenge was feasible in selected patients without universal recurrence of hypophysitis. Compared with CTLA-4 inhibitor–associated hypophysitis, which is more common and often accompanied by pituitary enlargement and multiple anterior pituitary hormone deficiencies, PD-1 inhibitor–related hypophysitis appears considerably rarer and exhibits a distinct clinical phenotype (10, 34). Rather than presenting with panhypopituitarism, pembrolizumab-associated cases most frequently manifest as isolated ACTH deficiency, often in the absence of radiographic pituitary enlargement (8, 35). Our findings reinforce this pattern and suggest that selective vulnerability of the corticotropic axis may represent a hallmark of PD-1–associated pituitary toxicity. This distinctive phenotype has important diagnostic implications, as clinicians may not initially suspect hypophysitis in the absence of imaging abnormalities or multiple hormonal deficits (36). The temporal pattern of onset observed in our cohort further supports the concept that PD-1–associated hypophysitis may develop insidiously rather than acutely. Most cases occurred within several months of therapy initiation, yet delayed presentations were also observed. This variability indicates that endocrine surveillance should not be limited to early treatment cycles but should continue throughout the duration of immunotherapy. The delayed and sometimes subtle presentation may reflect gradual immune-mediated destruction of corticotroph cells rather than fulminant inflammatory enlargement of the gland.

The mechanisms underlying pembrolizumab-induced hypophysitis remain incompletely understood. Unlike CTLA-4, PD-1 is not strongly expressed on normal pituitary cells, suggesting that indirect immune activation rather than direct antibody-mediated cytotoxicity may predominate (37). Proposed mechanisms include autoreactive T-cell activation, cross-reactive immune responses, molecular mimicry, and epitope spreading triggered by systemic immune stimulation (38). Genetic predisposition, baseline immune milieu, and tumor-specific antigen profiles may further modulate susceptibility to endocrine toxicity (21). The predominance of isolated ACTH deficiency raises the possibility that corticotroph cells possess unique antigenic features or are particularly vulnerable to immune-mediated injury in the context of PD-1 blockade (39).

Clinically, most cases represent secondary adrenal insufficiency with preserved mineralocorticoid function, as aldosterone secretion remains intact in central disease. Nevertheless, severe presentations such as adrenal crisis, profound hyponatremia, and hemodynamic instability demonstrate that central adrenal insufficiency can still be life-threatening (40). In our cohort, acute hypotension and metabolic disturbances were documented in a subset of patients, underscoring the importance of rapid biochemical assessment when symptoms arise. Because adrenal insufficiency may initially manifest with nonspecific constitutional complaints, diagnostic delay is common. Symptoms such as fatigue, anorexia, nausea, weight loss, dizziness, and hypotension frequently overlap with malignancy progression, infection, chemotherapy-related toxicity, or other irAEs (41). Furthermore, pituitary MRI may remain normal in a substantial proportion of patients, reducing reliance on imaging as a diagnostic tool. These findings emphasize that biochemical evaluation—particularly early morning cortisol and ACTH measurement—should be prioritized in symptomatic patients receiving PD-1 inhibitors (42). Glucocorticoid replacement remains the cornerstone of management. In most patients, physiologic hydrocortisone dosing results in rapid symptomatic improvement, supporting the concept that hormonal deficiency rather than mass effect drives the clinical presentation. High-dose immunosuppressive corticosteroids appear unnecessary in the absence of significant pituitary enlargement or compressive symptoms. Importantly, hypophysitis alone does not universally necessitate permanent discontinuation of pembrolizumab. Carefully selected patients may tolerate continuation or rechallenge of ICI therapy once endocrine deficiencies are adequately controlled. This approach is clinically relevant, as premature discontinuation of effective immunotherapy may compromise oncologic outcomes.

Endocrine recovery appears uncommon in pembrolizumab-induced hypophysitis. Most patients require long-term or lifelong glucocorticoid replacement, suggesting irreversible corticotroph damage rather than transient inflammatory suppression (12). Persistent ACTH deficiency has important implications for survivorship care, including patient education regarding stress-dose steroid administration, perioperative management, infection-related dose adjustment, and prevention of adrenal crisis (43). Structured follow-up and collaboration between oncologists and endocrinologists are therefore essential components of long-term management. The relationship between endocrine immune-related adverse events and antitumor efficacy remains an area of ongoing investigation. Some studies suggest that immune-mediated endocrinopathies may reflect enhanced systemic immune activation and potentially correlate with improved oncologic response (16, 44). Although our analysis was not designed to establish causality, the favorable tumor control observed in many reported cases raises the possibility that hypophysitis may serve as a surrogate marker of robust immune engagement. Future prospective studies integrating endocrine monitoring with immune profiling may clarify whether specific endocrine toxicities are linked to durable tumor responses.

### Limitations of the study

Several limitations should be acknowledged. As a retrospective synthesis of published case reports and small case series, this study is inherently subject to publication bias and reporting bias. Severe, unusual, or well-documented cases are more likely to be reported, whereas mild, asymptomatic, or rapidly resolved cases may be underrepresented, potentially leading to an underestimation of the true incidence. In addition, diagnostic criteria, endocrine evaluation, MRI protocols, laboratory sampling times, and follow-up duration varied across reports. Some pituitary axes were incompletely assessed, and longitudinal hormonal data were limited, which may affect the interpretation of endocrine phenotypes and recovery patterns. Nevertheless, given the rarity of pembrolizumab-induced hypophysitis, pooled analyses provide valuable insight into its clinical spectrum and practical management considerations.

## Conclusion

In summary, pembrolizumab-induced hypophysitis represents a rare but clinically meaningful endocrine irAE characterized predominantly by isolated ACTH deficiency, frequently normal pituitary imaging, low rates of hormonal recovery, and generally favorable symptomatic response to glucocorticoid replacement. Early recognition, ongoing endocrine surveillance, and individualized decisions regarding ICI continuation are critical to balancing oncologic efficacy with patient safety. As the indications for PD-1 blockade continue to expand, heightened awareness of this distinctive endocrine toxicity will be increasingly important in routine clinical practice.

## Data Availability

The original contributions presented in the study are included in the article/**Supplementary Material**. Further inquiries can be directed to the corresponding author.

## References

[1] Carlino MS Larkin J Long GV . Immune checkpoint inhibitors in melanoma. Lancet. (2021) 398:1002–14. doi: 10.1016/s0140-6736(21)01206-x 34509219

[2] Malmberg R Zietse M Dumoulin DW Hendrikx J Aerts J van der Veldt AAM . Alternative dosing strategies for immune checkpoint inhibitors to improve cost-effectiveness: A special focus on nivolumab and pembrolizumab. Lancet Oncol. (2022) 23:e552–61. doi: 10.1016/s1470-2045(22)00554-x 36455584

[3] Marchand A Kervarrec T Bhatia S Samimi M . Pembrolizumab and other immune checkpoint inhibitors in locally advanced or metastatic Merkel cell carcinoma: Safety and efficacy. Expert Rev Anticancer Ther. (2020) 20:1093–106. doi: 10.1080/14737140.2021.1835477 33044876

[4] Abulizi A Yan G Xu Q Muhetaer R Wu S Abudukelimu K . Cardiovascular adverse events and immune-related adverse events associated with Pd-1/Pd-L1 inhibitors for head and neck squamous cell carcinoma (Hnscc). Sci Rep. (2024) 14:25919. doi: 10.1038/s41598-024-75099-5 39472591 PMC11522629

[5] Baxi S Yang A Gennarelli RL Khan N Wang Z Boyce L . Immune-related adverse events for anti-Pd-1 and anti-Pd-L1 drugs: Systematic review and meta-analysis. Bmj. (2018) 360:k793. doi: 10.1136/bmj.k793 29540345 PMC5851471

[6] Fukushima T Kobayashi S Ueno M . The correlation between immune-related adverse events and efficacy of immune checkpoint inhibitors. Jpn J Clin Oncol. (2024) 54:949–58. doi: 10.1093/jjco/hyae067 38769817 PMC11374884

[7] Markovic M Niciforovic D Mladenovic V Pavlovic D Papic D Milojevic K . Immune-related adverse events-pembrolizumab-induced colitis-the importance of early diagnosis and treatment: A case report and review of the literature. Int J Immunopathol Pharmacol. (2025) 39:3946320251326699. doi: 10.1177/03946320251326699 40231646 PMC12033556

[8] Wright JJ Powers AC Johnson DB . Endocrine toxicities of immune checkpoint inhibitors. Nat Rev Endocrinol. (2021) 17:389–99. doi: 10.1038/s41574-021-00484-3 33875857 PMC8769055

[9] Barroso-Sousa R Barry WT Garrido-Castro AC Hodi FS Min L Krop IE . Incidence of endocrine dysfunction following the use of different immune checkpoint inhibitor regimens: A systematic review and meta-analysis. JAMA Oncol. (2018) 4:173–82. doi: 10.1001/jamaoncol.2017.3064 28973656 PMC5838579

[10] Darnell EP Mooradian MJ Baruch EN Yilmaz M Reynolds KL . Immune-related adverse events (Iraes): Diagnosis, management, and clinical pearls. Curr Oncol Rep. (2020) 22:39. doi: 10.1007/s11912-020-0897-9 32200442

[11] Faje A Reynolds K Zubiri L Lawrence D Cohen JV Sullivan RJ . Hypophysitis secondary to nivolumab and pembrolizumab is a clinical entity distinct from ipilimumab-associated hypophysitis. Eur J Endocrinol. (2019) 181:211–9. doi: 10.1530/eje-19-0238 31176301

[12] Montero Pérez O Sánchez Escudero L Guzmán Ramos MI Aviñó Tarazona V . Hypophysitis secondary to pembrolizumab: A case report and review of the literature. Anticancer Drugs. (2022) 33:94–9. doi: 10.1097/cad.0000000000001129 34261922

[13] Rossi S Silvetti F Bordoni M Ciarloni A Salvio G Balercia G . Pembrolizumab-induced thyroiditis, hypophysitis and adrenalitis: A case of triple endocrine dysfunction. JCEM Case Rep. (2024) 2:luae200. doi: 10.1210/jcemcr/luae200 39498471 PMC11532647

[14] Mohammed SS Alrosan S Asad R . Adrenal crisis due to pembrolizumab-induced hypophysitis in a patient with triple-negative breast cancer. Endocr Oncol. (2025) 5:e250046. doi: 10.1530/eo-25-0046 40584406 PMC12203771

[15] Leiter A Gnjatic S Fowkes M Kim-Schulze S Laface I Galsky MD . A common pituitary autoantibody in two patients with immune checkpoint inhibitor-mediated hypophysitis: Zcchc8. AACE Clin Case Rep. (2020) 6:e151–60. doi: 10.4158/accr-2019-0585 32671216 PMC7357610

[16] Mimura C Tachihara M Kusuhara S Fukuoka H Nishimura Y . Complete response in a patient with lung cancer suffering from three pembrolizumab-induced immune-related adverse events including retinal vasculitis. Respirol Case Rep. (2021) 9:e00730. doi: 10.1002/rcr2.730 33732464 PMC7938210

[17] Balti E Verhaeghe S Kruse V Roels S Coremans P . Exploring a new entity of single-agent pembrolizumab-associated hypophysitis. Cureus. (2022) 14:e27763. doi: 10.7759/cureus.27763 36127991 PMC9481187

[18] Do TVC Gudipati MK Ganti SS Depa J Sajnani K . Immunotherapy: A case series. Cureus. (2021) 13:e19726. doi: 10.7759/cureus.19726 34934589 PMC8684541

[19] Tanaka S Kushimoto M Nishizawa T Takubo M Mitsuke K Ikeda J . Isolated Acth deficiency during single-agent pembrolizumab for squamous cell lung carcinoma: A case report. Clin Diabetes Endocrinol. (2020) 6:1. doi: 10.1186/s40842-019-0092-9 31921440 PMC6945618

[20] Mishra T He G Sreeram K Rauf M Subahi A Hazem M . Immune checkpoint inhibitor-associated central adrenal insufficiency. Am J Ther. (2019) 26:e626–7. doi: 10.1097/mjt.0000000000000832 30277909

[21] Hinata Y Ohara N Sakurai Y Koda R Yoneoka Y Takada T . Isolated adrenocorticotropic hormone deficiency associated with severe hyperkalemia during pembrolizumab therapy in a patient with ureteral cancer and an ileal conduit: A case report and literature review. Am J Case Rep. (2021) 22:e931639. doi: 10.12659/ajcr.931639 34262010 PMC8297058

[22] Oğuz SH Ünlütürk U Aksoy S Erbas T . Clinical course and management of pembrolizumab-associated isolated adrenocorticotrophic hormone deficiency: A new case and literature review. Immunotherapy. (2021) 13:1157–63. doi: 10.2217/imt-2021-0061 34387129

[23] Than CM Maung KM Win Y Htwe N . Beyond the tumour: An endocrine pitfall of immunotherapy. Cureus. (2025) 17:e91766. doi: 10.7759/cureus.91766 41069905 PMC12504602

[24] Kethireddy N Thomas S Bindal P Shukla P Hegde U . Multiple autoimmune side effects of immune checkpoint inhibitors in a patient with metastatic melanoma receiving pembrolizumab. J Oncol Pharm Pract. (2021) 27:207–11. doi: 10.1177/1078155220921543 32390537

[25] Fernando A Mittal A Cheema R . Nothing great comes without its risks: A rare case of pembrolizumab-induced hypophysitis. JCEM Case Rep. (2024) 2:luad135. doi: 10.1210/jcemcr/luad135 38116159 PMC10729852

[26] Shah N Prentice D Jayasundera C . Pd-1 inhibitor-induced thyroiditis and Acth deficiency. BMJ Case Rep. (2025) 18:e254034. doi: 10.1136/bcr-2022-254034 40000036

[27] Khan WJ Asif M Chaudhry HS Aslam S Nadeem I . Pembrolizumab-induced broken heart syndrome, pneumonitis, and hypophysitis occurring concurrently; a deadly triad. J Community Hosp Intern Med Perspect. (2023) 13:88–90. doi: 10.55729/2000-9666.1173 37877053 PMC10593175

[28] Ogbodu K Sharda M Vanani NB Jha P . Pembrolizumab-induced hypophysitis in the setting of renal cell carcinoma. Cureus. (2025) 17:e82940. doi: 10.7759/cureus.82940 40416222 PMC12103938

[29] Perera J Bhanot S . Pembrolizumab-induced hypothyroidism and diabetes mellitus: A rare case presentation post-treatment. Cureus. (2025) 17:e84880. doi: 10.7759/cureus.84880 40575214 PMC12199207

[30] Doodnauth AV Klar M Mulatu YS Malik ZR Patel KH McFarlane SI . Pembrolizumab-induced hypophysitis with isolated adrenocorticotropic hormone (Acth) deficiency: A rare immune-mediated adverse event. Cureus. (2021) 13:e15465. doi: 10.7759/cureus.15465 34123679 PMC8186842

[31] Al Heyasat A Chaudhry MS Alkharabsheh M Bani Amer M Poojary I . Pembrolizumab-induced hypophysitis: A rare immune-related adverse event in a patient with metastatic non-small cell lung cancer. Cureus. (2025) 17:e82701. doi: 10.7759/cureus.82701 40400852 PMC12094803

[32] Webb MJ Breen WG Laack NN Leventakos K Campian JL Sener U . Proton craniospinal irradiation with bevacizumab and pembrolizumab for leptomeningeal disease: A case report. CNS Oncol. (2023) 12:Cns101. doi: 10.2217/cns-2023-0005 37491842 PMC10410687

[33] Onoyama I Kawakami M Hachisuga K Maenohara S Kodama K Yagi H . Secondary adrenal insufficiency due to isolated Acth deficiency induced by pembrolizumab: A report of two cases of uterine endometrial cancer. Rep (MDPI). (2023) 6:18. doi: 10.3390/reports6020018 40729185 PMC12225413

[34] Chang LS Barroso-Sousa R Tolaney SM Hodi FS Kaiser UB Min L . Endocrine toxicity of cancer immunotherapy targeting immune checkpoints. Endocr Rev. (2019) 40:17–65. doi: 10.1210/er.2018-00006 30184160 PMC6270990

[35] Khoja L Day D Wei-Wu Chen T Siu LL Hansen AR . Tumour- and class-specific patterns of immune-related adverse events of immune checkpoint inhibitors: A systematic review. Ann Oncol. (2017) 28:2377–85. doi: 10.1093/annonc/mdx286 28945858

[36] Spain L Diem S Larkin J . Management of toxicities of immune checkpoint inhibitors. Cancer Treat Rev. (2016) 44:51–60. doi: 10.1016/j.ctrv.2016.02.001 26874776

[37] Oliveira C Mainoli B Duarte GS MaChado T Tinoco RG Esperança-Martins M . Immune-related serious adverse events with immune checkpoint inhibitors: Systematic review and network meta-analysis. Eur J Clin Pharmacol. (2024) 80:677–84. doi: 10.1007/s00228-024-03647-z 38372756 PMC11001692

[38] Kanie K Iguchi G Bando H Urai S Shichi H Fujita Y . Mechanistic insights into immune checkpoint inhibitor-related hypophysitis: a form of paraneoplastic syndrome. Cancer Immunol Immunother. (2021) 70:3669–77. doi: 10.1007/s00262-021-02955-y 33977343 PMC8571153

[39] Mizukoshi T Fukuoka H Takahashi Y . Immune checkpoint inhibitor-related hypophysitis. Best Pract Res Clin Endocrinol Metab. (2022) 36:101668. doi: 10.1016/j.beem.2022.101668 35562229

[40] Gougis P Jochum F Abbar B Dumas E Bihan K Lebrun-Vignes B . Clinical spectrum and evolution of immune-checkpoint inhibitors toxicities over a decade-a worldwide perspective. EClinicalMedicine. (2024) 70:102536. doi: 10.1016/j.eclinm.2024.102536 38560659 PMC10981010

[41] Di Stasi V La Sala D Cozzi R Scavuzzo F De Geronimo V Poggi M . Immunotherapy-related hypophysitis: A narrative review. Cancers (Basel). (2025) 17:436. doi: 10.3390/cancers17030436 39941803 PMC11815778

[42] Johnson J Goldner W Abdallah D Qiu F Ganti AK Kotwal A . Hypophysitis and secondary adrenal insufficiency from immune checkpoint inhibitors: Diagnostic challenges and link with survival. J Natl Compr Canc Netw. (2023) 21:281–7. doi: 10.6004/jnccn.2022.7098 36828029

[43] Zhou Z Wang H . Successful rechallenge of immune checkpoint inhibitors after severe immune-related hepatitis, thyroiditis and hypophysitis in Tmb-high Nsclc: A case report. Transl Lung Cancer Res. (2025) 14:2875–9. doi: 10.21037/tlcr-2025-341 40799443 PMC12337046

[44] Jessel S Weiss SA Austin M Mahajan A Etts K Zhang L . Immune checkpoint inhibitor-induced hypophysitis and patterns of loss of pituitary function. Front Oncol. (2022) 12:836859. doi: 10.3389/fonc.2022.836859 35350573 PMC8958012

